# Microplastic exposure and allergic rhinitis: Network toxicology, and molecular docking insights

**DOI:** 10.1371/journal.pone.0334162

**Published:** 2025-10-17

**Authors:** Yaojun Wang, Dandan Xu

**Affiliations:** 1 Affiliated Hospital, Clinical Medical College, Hebei University, Baoding, Hebei, China; 2 Hebei Key Laboratory of Systems Biology and Gene Regulation, The First Affiliated Hospital of Hebei North University, zhangjiakou, China; 3 Central Laboratory, The First Affiliated Hospital of Hebei North University; Kazi Nazrul University, INDIA

## Abstract

**Background:**

Microplastics (MPs), ubiquitous environmental pollutants, are increasingly associated with global health risks, yet their role in allergic rhinitis (AR) pathogenesis remains poorly understood.

**Methods:**

Toxicity profiles of four typical MPs (polyethylene [PE], polypropylene [PP], polyvinyl chloride [PVC], polystyrene [PS]) were evaluated using ADMETlab 3.0. MP-related targets and AR-associated genes were integrated from the CTD database and GSE43523 dataset. Functional enrichment (GO/KEGG) and PPI network analysis (STRING/GeneMANIA) were performed on overlapping genes. LASSO regression and expression validation identified key targets, and molecular docking (Autodock Vina) assessed interactions with potential therapeutics predicted by CTD.

**Results:**

ADMET analysis revealed MPs exhibit significant respiratory toxicity and ocular toxicity. We identified 301 MP toxicity targets, 1,026 AR differentially expressed genes (DEGs), and 15 overlapping pathogenic targets. Functional enrichment (GO/KEGG) demonstrated MPs disrupt respiratory mucosal homeostasis via apoptosis, mitochondrial autophagy, and inflammatory pathways. PPI network analysis and LASSO regression pinpointed DNAJB9, SQSTM1, and MAPK9 as core mediators: these genes were significantly downregulated in AR patients (P < 0.05) and displayed robust diagnostic performance (AUC = 0.82–0.93). Molecular docking revealed resveratrol binds these targets with high affinity, surpassing SQSTM1 (−5.8 kcal/mol) and MAPK9 (−6.8 kcal/mol), suggesting its potential to block MP-induced dysregulation.

**Conclusions:**

MPs drive AR pathogenesis through respiratory toxicity pathways, with DNAJB9, SQSTM1, and MAPK9 serving as critical molecular mediators. Resveratrol, by modulating target-mediated programmed cell death, emerges as a promising therapeutic candidate for mitigating MP-induced AR.

## 1 Introduction

MPs have received a great deal of attention as an emerging environmental pollutant due to their widespread presence and potential health risks [1,2]. These particles, which are less than 5 millimetres in diameter, originate primarily from the physicochemical degradation of plastic products, emissions from industrial production and the disposal of discarded consumer goods. Since Thompson et al. formally defined the concept in 2004 [3], there has been a significant positive correlation between global microplastic pollution levels and thermoplastic production. The primary sources are PE, PP, PS, PVC and PET [4]. Research indicates that global annual thermoplastic production has surpassed 400 million tonnes, and this sustained growth in plastic manufacturing is recognised as a key driver of escalating microplastic pollution [5–7]. While the exact conversion rate between plastic production and environmental microplastic release is unclear, the scientific community broadly agrees that the exponential increase in plastic production is a primary cause of the global microplastic pollution crisis [6]. Notably, microplastic pollution exhibits multi-medium characteristics, spanning soil, freshwater and marine ecosystems, the atmosphere, drinking water sources and even human tissues. Its contamination trajectory now encompasses the entire biogeochemical cycle [8–10].

The detection of MPs in air is chiefly dependent on the utilisation of sophisticated sampling and analytical techniques, including high-flow air samplers, impactor samplers, and wet deposition sampling. These methods have been demonstrated to be effective in the capture and identification of plastic particles across a wide size range (typically <5 μm) [11]. Recent epidemiological evidence indicates that AMPs can enter the human body via the respiratory tract, thereby posing a significant threat to respiratory health [2,12,13]. One study analysed venous blood samples from 22 non-fasting adult volunteers, detecting multiple MPs with concentrations as high as 7.1 μg/mL for a single type. In addition, related research has confirmed the presence of MPs in human placentas [14,15]. Prata’s research estimates that the average person inhales approximately 26–130 microplastic particles daily via the respiratory tract [16], with nasal exposure potentially disrupting the body’s energy metabolism balance [17]. In this context, AR, an IgE-mediated chronic inflammatory disease, has emerged as a research focus. The impact of AR on patients’ quality of life is significant, with negative effects on socialisation, education, and work, as well as substantial economic burdens [18]. The typical symptoms associated with this condition include paroxysmal sneezing, watery nasal discharge, nasal congestion, and nasal itching [19–21]. Its pathogenesis is closely associated with environmental exposures, particularly classic allergens such as pollen, dust mites, tobacco smoke, and pet dander [22]. Despite the recent emergence of studies exploring the potential correlation between microplastic exposure and the onset and progression of AR, the specific mechanisms underpinning this relationship remain to be fully elucidated.

Early exploratory studies have detected microplastic particles in patients with AR [12,23]. Among these, a prospective cohort study involving 66 participants (36 AR patients + 30 healthy controls) analyzed nasal samples and first confirmed microplastic accumulation in the nasal environment of AR patients [23]. Another cross-sectional study involving 33 AR patients and 22 healthy controls focused on nasal lavage fluid samples, further validating the specific distribution characteristics of MPs in the AR population [12]. These findings suggest that MPs may enter the human body through multiple pathways, including inhalation of polluted air, ingestion of contaminated food/water, and skin contact. Among these, the respiratory tract—as a mucosal interface directly connected to the atmospheric environment—is considered one of the key routes for microplastic exposure [1,24].

Network toxicology is an emerging research methodology grounded in the principles of network pharmacology and network biology. The integration of techniques such as bioinformatics, big data analysis, genomics, proteomics and metabolomics is a key feature of the methodology [25]. Network toxicology is an emerging research methodology grounded in the principles of network pharmacology and network biology. The integration of methodologies encompasses bioinformatics, big data analysis, genomics, proteomics and metabolomics.The present study aims to employ network toxicology, bioinformatics and molecular docking techniques to elucidate the potential toxicity mechanisms of MPs, building upon this methodological framework. The molecular pathways and target interactions linking MPs to ARwill be investigated.

## 2 Materials and methods

### 2.1 Data collection related to MPs

**Fig 1:**
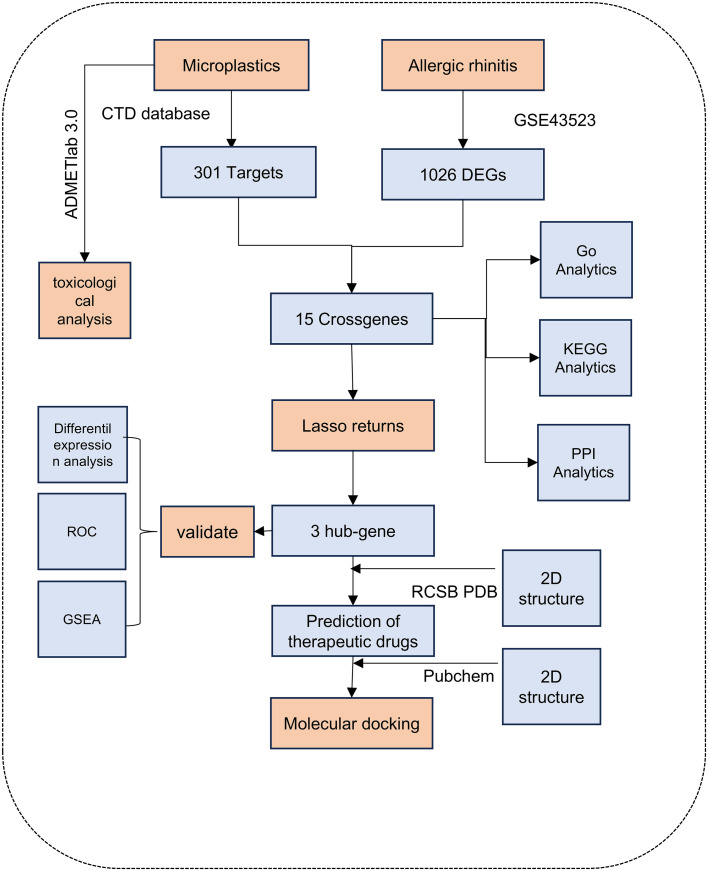
Workflow of the analysis.

The design methodology and flow of this study is demonstrated in Fig 1.In this study, the toxicological properties of MPs (PE, PP, PVC, and PS) were evaluated using ADMETlab 3.0 (https://admetmesh.scbdd.com/) [26]. Subsequently, the targets of action related to MPs were retrieved from the CTD (Comparative Toxicogenomics Database) database [27]. Furthermore, the 2D molecular structures of PE, PP, and PVC were obtained through PubChem (https://pubchem.ncbi.nlm.nih.gov/) to provide fundamental data for subsequent research analysis [28]. Finally, an interactive network was constructed using Cytoscape 3.8.2 software to visualise the relationship between these components and targets.

### 2.2 AR-related target acquisition and differential expression analysis

The GEO dataset (GSE43523) used in this study comes from the NCBI public database (https://www.ncbi.nlm.nih.gov/gds),). The original research has passed the corresponding ethical review (see the ‘ Methods ‘ section of the dataset for specific information).A comprehensive search of the GEO database was conducted, with a focus on datasets involving the species “Homo sapiens”. The selection criteria included studies pertaining to nasal epithelial cells, with a particular emphasis on samples from both healthy volunteers and patients afflicted with seasonal AR. The dataset GSE43523 comprises samples from six patients diagnosed with seasonal ARand an equivalent number of healthy controls. Subsequently, the differentially expressed genes were screened by the “limma” package in R in order to identify significant differences between patients and healthy controls. In this process, invalid gene symbols or genes with multiple probe sets were removed and averaged. The statistical significance of the findings was determined by a p-value < 0.05. In volcano plots and analysis of variance scatter plots, the distribution of up- and down-regulated genes in patients with ARis demonstrated.

### 2.3 Intersection between MPs and AR

We matched AR targets with MPs action targets and made a Wayne diagram using the Venny 2.1 online tool. The overlapping ones (intersections) in the Wayne diagram show possible targets of MPs in allergic rhinitis. Also, we made a grouped heat map of the crossgenes using ImageGP [29] to visualize the expression patterns of these genes.

### 2.4 Enrichment analysis

To study the functions of the crossgenes, we did gene ontology (GO) and Kyoto Encyclopedia of Genes and Genomes (KEGG) pathway enrichment tests on these genes. We used R tools (clusterProfiler and enrichplot) [30]. We considered a P value below 0.05 as meaningful. We also used bubble plots to show the GO results and chordal plots to display the KEGG results..

### 2.5 Protein-protein network analysis

To study how the crossgenes interact with each other, we built a PPI network for potential MPs targets related to ARusing the STRING database (https://stringdb.org/) [31]. We set the organism to “Homo sapiens” and used a medium confidence level (0.400) for interactions. Then we ran MCL clustering analysis through the STRING database to examine relationships between the overlapping genes. We also used the Genemama database to analyze functional networks of these targets and find possible signaling pathways.

### 2.6 Microplastic-target-pathway network

We used Cytoscape software to build a network showing connections between MPs, their targets, and related pathways in allergic rhinitis.

### 2.7 Predictive model construction and evaluation

To find key MPs targets in allergic rhinitis, we organized gene expression data from the overlapping targets and ran Lasso regression analysis using R’s “glmnet” package. Lasso regression helps prevent overfitting and improves model performance by using an L1 penalty to select important variables. We grouped the clinical data and created heatmaps with the key genes to see how the regression model related to gene expression. We also used ROC analysis to check how well the regression model could diagnose the condition.

### 2.8 Internal validation of key genes

To test how well key genes can diagnose AR, we first checked if these genes had different expression levels in AR patients using non-parametric tests. We showed the results with violin plots. Next, we used ROC curve analysis to confirm how well these genes could identify AR. We also ran GSEA (Gene Set Enrichment Analysis) [31] to find which specific signaling pathways were connected to these key genes in AR [32].

### 2.9 Prediction and molecular docking of key target-therapeutics

To find possible drugs for DNAJB9, SQSTM1, and MAPK9, we searched the CTD database for small-molecule compounds. We then built a “key gene–small-molecule drug” network using Cytoscape. By using Venn diagrams, we found small-molecule drugs that work on all three genes and identified one natural small-molecule compound as a potential treatment. We got the 3D structures of the target proteins from the RCSB PDB database [33], Using PyMOL, we removed water molecules and original ligands. We then used AutoDock Tools 1.5.7 to add hydrogens, calculate charges, and adjust nonpolar hydrogen bonds. At the same time, we used Open Babel to change the drug’s.sdf file to PDB format. After setting the grid box size and genetic algorithm parameters, we ran molecular docking with AutoDock Vina. We used PyMOL to show the docking results, checking how well the target proteins bind to the natural drug and where they interact. These steps help support future drug development.

## 3 Results

### 3.1 Preliminary assessment of microplastic toxicity

**Fig 2:**
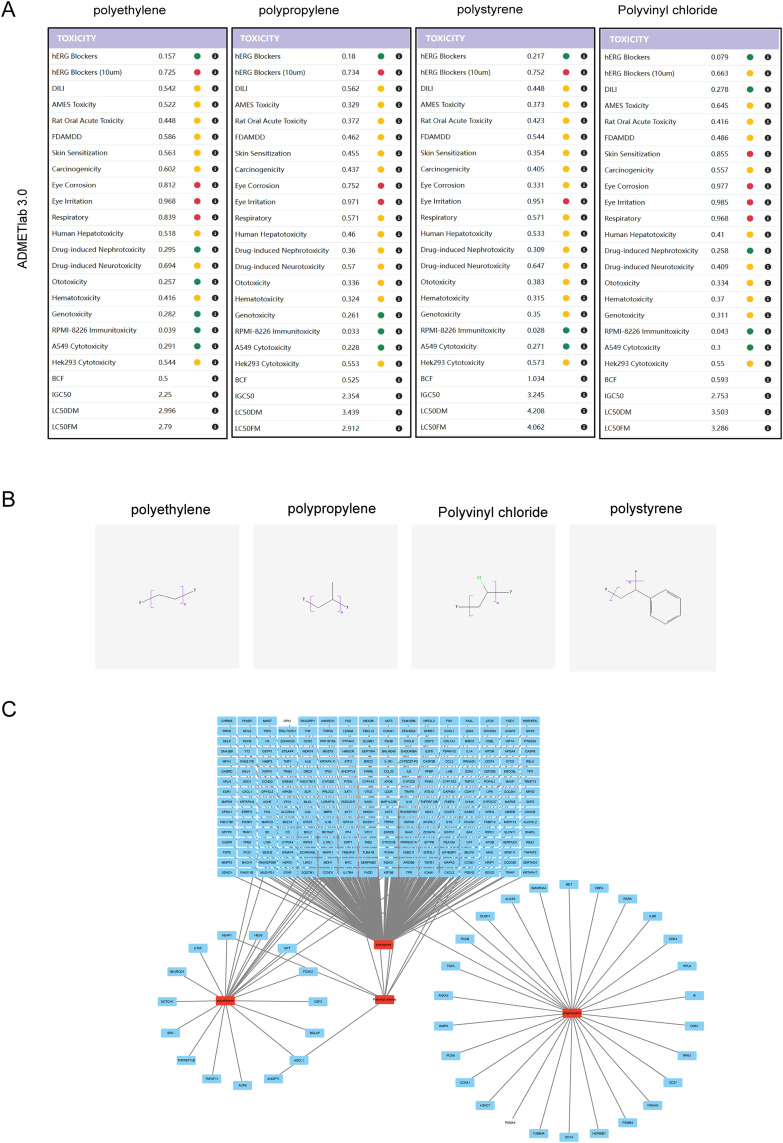
A. A heat map of toxicity endpoints is generated based on the ADMETlab 3.0 platform. Red, yellow, and green represent high risk (>0.7), medium risk (0.3–0.7), and low risk (<0.3), respectively. The toxicity scores of each polymer are numerically marked to show the relative risk level. **B.** 2D structures of polyethylene, polypropylene, polyvinyl chloride, and polystyrene. **C.**Microplastic-target network diagram. Red squares represent microplastics and blue squares represent corresponding targets.

The systematic assessment of environmental and health toxicity endpoints for four representative polymers—PE, PP, PVC, and PS—was conducted using the ADMETlab 3.0 platform. Results indicate (Fig 2A), For hERG blockade activity, polystyrene scored highest (0.217) with significantly elevated risk at 10 μM (0.752, red alert), while polyvinyl chloride scored lowest (0.079, 0.663 at 10 μM, yellow alert). For developmental and reproductive toxicity, polyethylene and polypropylene exhibited similar risks in DILI (0.542 and 0.562, yellow) and AMES (0.522 and 0.329, yellow) indicators, while polystyrene showed slightly higher risks in AMES (0.373, yellow) and carcinogenicity (0.405, yellow), while polyvinyl chloride was safest in DILI (0.278, green). In acute and chronic toxicity, polyvinyl chloride showed significantly higher risks than others in skin sensitization (0.855, red), eye irritation (0.985, red), and respiratory tract irritation (0.968, red). Regarding specific target organ toxicity, polystyrene and polyvinyl chloride exhibit higher risks for neurotoxicity (0.647 and 0.409, yellow, respectively); The above findings indicate that polyvinyl chloride poses a prominent risk for skin/respiratory sensitization, polystyrene warrants attention for hERG blockade risk, while polyethylene and polypropylene exhibit overall lower toxicity but still require consideration for eye irritation and other factors.

### 3.2 Identify the toxic targets induced by MPs and AR related targets

To identify toxicity targets related to MPs, we first obtained the 2D structures of MPs from the PubChem database (Fig 2B). According to the CTD database, PE has 23 targets, PP has 28, PVC has 9, and PS has 256. After integration and deduplication, we ultimately identified 301 potential microplastic-related toxicity targets. Finally, we constructed a microplastic-toxicity target network using Cytoscape software (Fig 2C), with the specific target list provided in Supplementary S1 Table.

**Fig 3:**
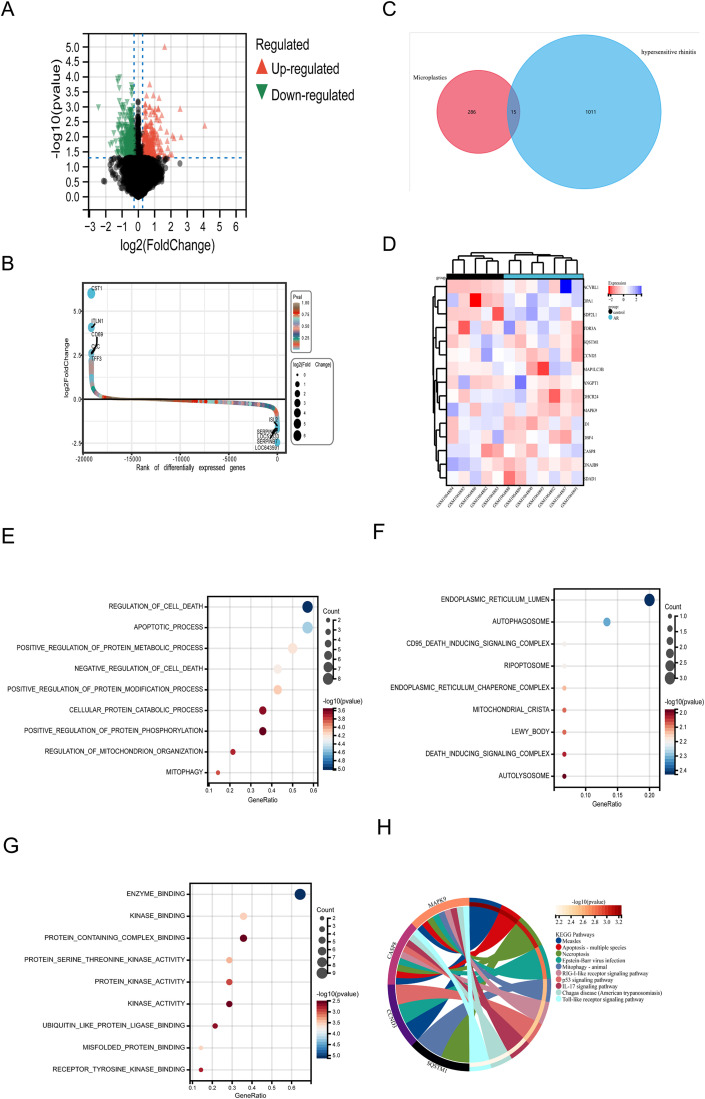
Screening of differentially expressed genes and functional enrichment analysis of crossover genes in allergic rhinitis. **A.**The red and green colors indicate significantly upregulated and downregulated genes. **B.**Scatter plot of differentially expressed genes in AR. **C.**The Venn diagram illustrates the intersection of genes between microplastic toxicity targets and differentially expressed genes for AR. **D.**The clustered heatmap shows the expression profile data of the 15 intersecting genes in AR. Red color represents gene expression upregulation and blue color represents gene expression downregulation. **E-G.** Biological process (BP), cellular composition (CC), and molecular function (MF) were presented using bubble plots. The size of the bubbles represents the number of enriched genes, and the color represents the significance. **H.** KEGG analysis results were presented using string diagrams, with different colors indicating different significant pathways and their associated genes.

We screened 1,026 DEGs related to AR from the GSE43523 dataset and visualized them using a volcano plot (Fig 3A). Among these genes, 461 were upregulated and 565 were downregulated. Fig 3B presents the overall distribution of the differentially expressed genes through a scatter plot, with the proportions of upregulated and downregulated genes labeled.

### 3.3 Identifying MPs and AR shared targets and functional enrichment

The intersection of microplastic toxicity targets and AR targets was analyzed using a Venn diagram, 15 shared pathogenic targets (Fig 3C, Supplementary S2 Table). After compiling the expression profiles of these genes, a heatmap was generated to visualize the results, where red indicates upregulated genes and green indicates downregulated genes (Fig 3D).

The biological functions of the intersecting genes were analyzed using GO and KEGG analyses. Function enrichment results revealed: Biological Processes (BP) primarily involved “Regulation of Cell Death,” “Apoptotic Process,” and “Positive Regulation of Protein Phosphorylation” (Fig 3E); Cellular Components (CC) were mainly enriched in “Endoplasmic Reticulum Lumen,” “Autophagosome,” and “Death Inducing Signaling Complex” (Fig 3F); Molecular Function (MF) was concentrated in “Enzyme Binding” and “Kinase Binding” (Fig 3G). KEGG analysis indicated that the intersecting genes were significantly enriched in “Measles,” “Apoptosis - multiple species,” and “Mitophagy - animal” (Fig 3H, Table 1). These intersecting targets may influence AR development by regulating apoptosis and autophagy mechanisms.

**Table 1:** KEGG enrichment analysis.

Description	pvalue	geneID
Measles	0.000569151	CASP8/MAPK9/CCND3
Apoptosis – multiple species	0.000698554	CASP8/MAPK9
Necroptosis	0.00090902	SQSTM1/CASP8/MAPK9
Epstein-Barr virus infection	0.001697599	CASP8/MAPK9/CCND3
Mitophagy – animal	0.002864927	SQSTM1/MAPK9
RIG-I-like receptor signaling pathway	0.003315143	CASP8/MAPK9
p53 signaling pathway	0.003503966	CASP8/CCND3
IL-17 signaling pathway	0.005782098	CASP8/MAPK9
Chagas disease (American trypanosomiasis)	0.006919876	CASP8/MAPK9
Toll-like receptor signaling pathway	0.007185568	CASP8/MAPK9

### 3.4 PPI network analysis and MPs-target-KEGG network

**Fig 4:**
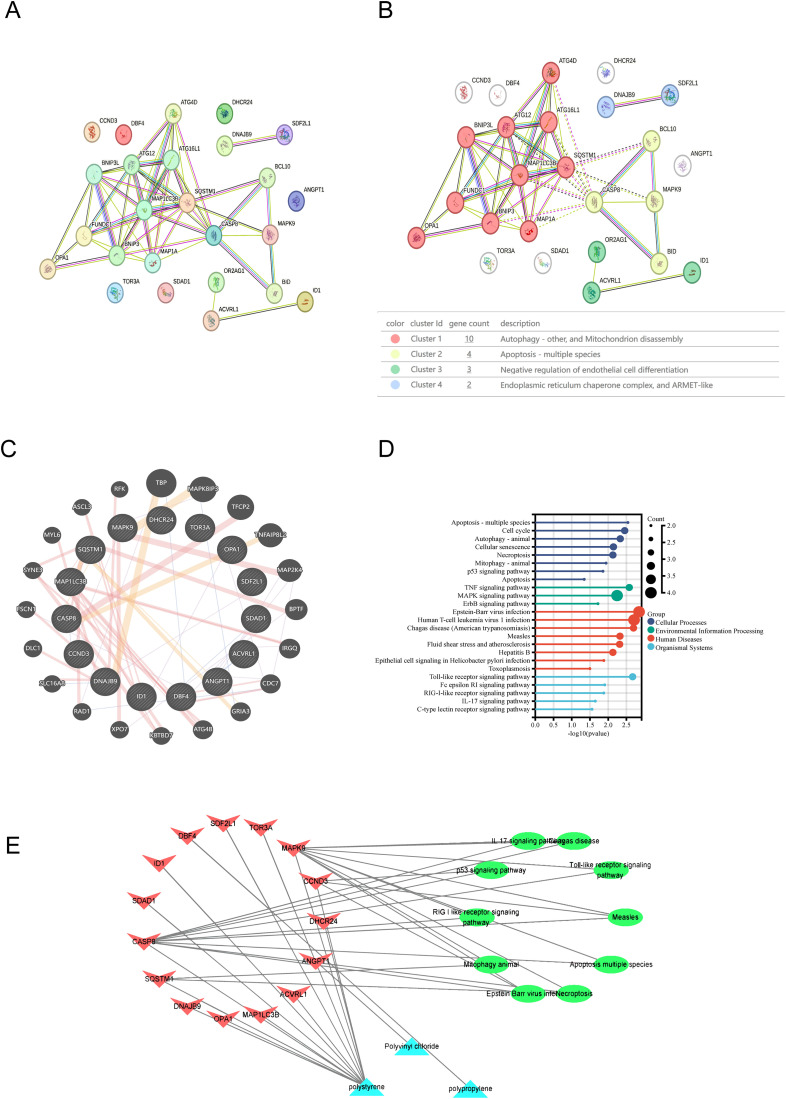
Protein-protein network analysis. **A.** The PPI network of intersecting genes is shown based on the STRING database. Circles represent gene nodes, and the frequency of connecting lines indicates the degree of interaction. **B.** The MCL clustering network of intersecting genes was demonstrated based on the STRING database. Different colored circles represent different clusters.**C.** PPI network of intersecting genes is shown based on the Genemama website. The inner black circles represent intersecting gene nodes and the outer black circles represent predicted gene nodes. The frequency of connecting lines indicates the degree of interaction.**D.** Demonstration of functional enrichment of intersecting genes in the Genemama database. Different colors represent different biological functions, and the size of the circle represents the number of genes enriched. **E.** Microplastic-target-pathway network. Blue triangles represent microplastics, red inverted triangles represent targets, and green ovals represent pathways.

Protein-protein interaction (PPI) networks for intersecting genes were constructed using the String database, 25 nodes and 53 edges with an average node degree of 4.24 (Fig 4A). MCL clustering analysis (Fig 4B) revealed that the intersecting genes clustered in pathways such as “Autophagy - other, and Mitochondrion disassembly,” “Apoptosis - multiple species,” “Negative regulation of endothelial cell differentiation,” and “Endoplasmic reticulum chaperone complex, and ARMET-like,” consistent with the functional enrichment analysis results.

GeneMANIA tool predicted 20 functionally similar genes (Fig 4C). Central genes are positioned in the inner circle, while predicted genes occupy the outer circle. Functional analysis revealed that most genes were associated with cell death-related pathways including “Apoptosis - multiple species,” “Autophagy - animal,” “Cell cycle,” and “Cellular senescence.” Environmental information primarily involved inflammatory pathways such as “TNF signaling pathway,” “MAPK signaling pathway,” and “ErbB signaling pathway.” human diseases focused on inflammatory conditions like “Epstein-Barr virus infection” and “Human T-cell leukemia virus 1 infection”; biological systems involved inflammatory responses such as “Toll-like receptor signaling pathway,” “Fc epsilon RI signaling pathway,” and “RIG-I-like receptor signaling pathway” (Fig 4D). These intersecting genes primarily promote AR progression by regulating cell death-related pathways.

To show the relationships among MPs, AR, and KEGG signaling pathways, we constructed a MPs-target-pathway network using Cytoscape software (Fig 4E). This network comprises 28 nodes and 38 edges. Results indicate that polystyrene has the highest number of targets, while CASP8 and MAPK9 are enriched in the most pathways.

### 3.5 Predictive model construction and verification

**Fig 5:**
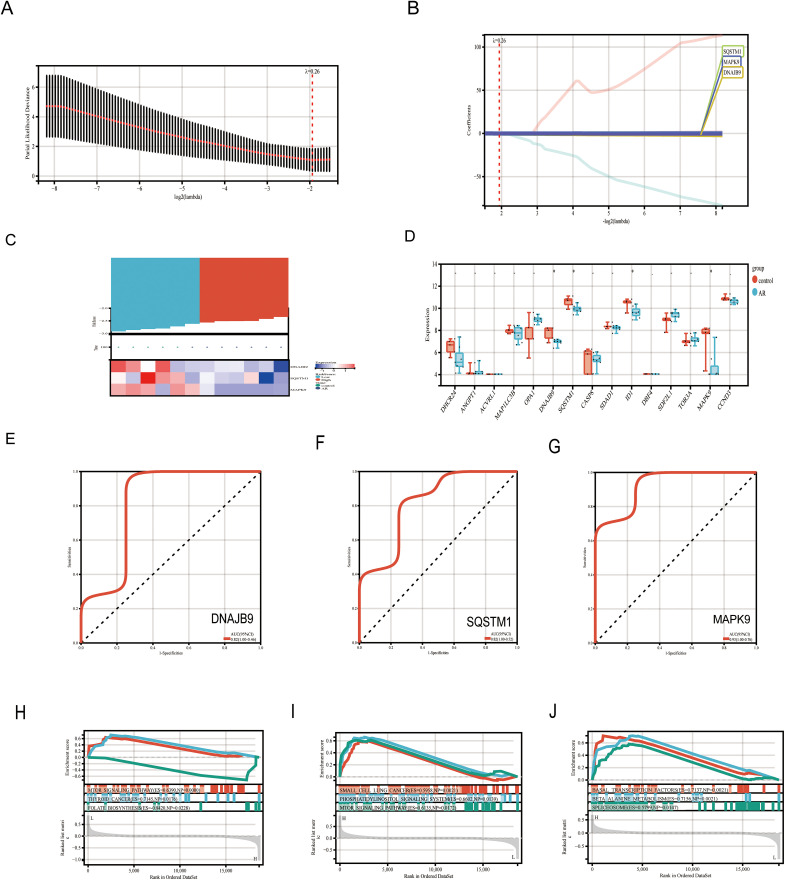
LASSO regression screening of key targets and their internal validation analysis. **A-B.** Lasso regression was used to screen key targets, and 3 of them were identified as key targets of microplastics acting in AR. **C.** Prognostic heatmap demonstrating the expression profile data of 3 key targets in AR. The upper part of it represents the risk score, where red color represents high risk and blue color represents low risk. The lower part represents the differential expression profiles of the genes. Blue color represents gene expression down-regulation and red color represents gene expression up-regulation. **D.** Box plot demonstrating the differential expression of 15 intersecting genes between AR and normal controls. *p < 0.05. **E-G.** ROC curves validate the diagnostic efficacy of the 3 key targets. **H-J.** Single-gene GSEA analysis of the 3 key targets. The upper part represents the enrichment score, the middle part represents the enrichment pathway, and the lower part represents the risk Rank.

Together with the risk factors identified by the Lasso linear regression analysis, we selected three key genes (DNAJB9, SQSTM1, and MAPK9) involved in the microplastic-induced AR pathway as risk factors (Fig 5A–5B). Phenotypic heatmap analysis revealed that DNAJB9, SQSTM1, and MAPK9 were all downregulated in AR patients compared to healthy individuals and were associated with higher risk (Fig 5C).

To further validate the diagnostic value of DNAJB9, SQSTM1, and MAPK9 in AR, we conducted differential expression analysis, ROC curve analysis, and GSEA analysis. DEGs revealed (Fig 5D) that DNAJB9, SQSTM1, and MAPK9 all exhibited a downward trend in AR patients, with P < 0.05 indicating statistical significance. ROC curve analysis results (Fig 5E–5G, Table 2) showed that the area under the curve (AUC) for DNAJB9 was 0.82 (95% CI: 1.0-0.46), SQSTM1 had an AUC of 0.82 (95% CI: 1.0–0.52), and MAPK9 had an AUC of 0.93 (95% CI: 1.0–0.76), all demonstrating good diagnostic performance. These findings suggest that DNAJB9, SQSTM1, and MAPK9 may serve as key targets for MPs influencing the progression of AR disease.

**Table 2:** ROC curve analysis of AUC and 95% CI for DNAJB9, SQSTM1, and MAPK9.

Gene	AUC	95% Confidence Interval (CI)
DNAJB9	0.82	0.76–0.86
SQSTM1	0.81	0.75–0.85
MAPK9	0.83	0.77–0.87

We further employed GSEA to explore the functional pathways enriched by genes, revealing that DNAJB9 was significantly enriched in the mTOR signaling pathway (ES = 0.639, NES = 1.49, p = 0.0000), thyroid cancer (ES = 0.714, NES = 1.65, p = 0.0176), and folate biosynthesis (ES = 0.852, NES = 1.60, p = 0.0228)(Fig 5H). SQSTM1 showed enrichment in small cell lung cancer (ES = 0.598, NES = 1.50, p = 0.0021), phosphatidylinositol signaling system (ES = 0.607, NES = 1.39, p = 0.0139), mTOR signaling pathway (ES = 0.617, NES = 1.37, p = 0.0172), among others(Fig 5I). MAPK9 was primarily enriched in pathways including basic transcription factors (ES = 0.713, NES = 1.67, p = 0.0021), β-alanine metabolism (ES = 0.715, NES = 1.65, p = 0.0021), and proteasome (ES = 0.579, NES = 1.47, p = 0.0147) pathways(Fig 5J). This suggests that these three genes may participate in relevant biological processes or disease progression by regulating multiple key biological pathways.

### 3.6 Prediction and molecular docking of therapeutic agents for key toxicity targets

**Fig 6:**
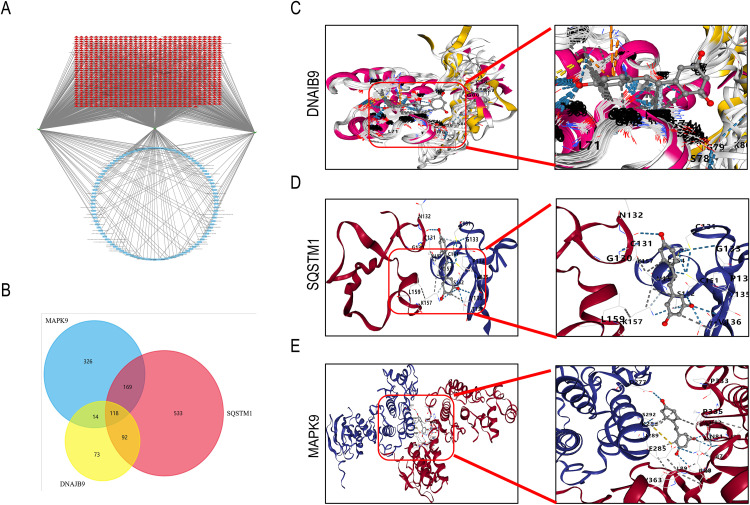
Prediction of therapeutic drugs for key targets and molecular docking. A. Prediction of therapeutic drugs for key targets based on the CTD database and presented as a key target-therapeutic drug network. Where green squares represent key targets, red squares represent therapeutic drugs, and blue squares represent therapeutic drugs shared by 3 targets.B. 118 shared therapeutic drugs were obtained by Wayne diagrams. C-E. Molecular docking results of the 3 key targets with resveratrol, respectively.

To identify small-molecule drugs associated with DNAJB9, SQSTM1, and MAPK9, we searched the CTD database. Results showed that DNAJB9 predicted 228 drugs, SQSTM1 predicted 706 drugs, and MAPK9 predicted 474 drugs. Based on these findings, we constructed a key target-small-molecule drug network (Fig 6A). Through Venn diagram analysis, we identified 118 compounds common to all three genes (Fig 6B). Literature review indicated that resveratrol [34–38], among these 118 compounds, may mitigate microplastic toxicity damage and serves as a potential therapeutic agent.

The results of molecular docking indicate that resveratrol can form stable binding conformations with core target proteins (DNAJB9, SQSTM1, MAPK9). Binding affinity data (Fig 6C–6E, Table 3) further validate its strong interaction characteristics: The binding energy between resveratrol and DNAJB9 reached −94.8 kcal/mol (significantly below the high-affinity threshold of −5 kcal/mol), while the binding energies with SQSTM1 (−5.8 kcal/mol) and MAPK9 (−6.8 kcal/mol) also met the criteria for high affinity. Key interaction sites are concentrated within the active pockets of the proteins, indicating that resveratrol forms highly stable bonds by occupying core functional regions of the targets. These findings suggest that the low binding energies (highly stable bonds) of resveratrol with the three proteins effectively block microplastic-induced abnormal signaling pathways, providing a molecular basis for its potential as a therapeutic agent for microplastic-related AR.

**Table 3:** Molecular docking of resveratrol with DNAJB9, SQSTM1, and MAPK9.

	ID Cavities	Vina score	volume center	x center	y center	z size
SQSTM1	1	−5.8	232	146.743	176.343	232.893
	2	−5.4	90	155.104	170.043	237.257
	3	−4.8	33	144.543	161.249	242.341
	4	−4.8	29	151.824	161.801	228.781
	5	−4.4	15	154.32	182.612	238.982
DNAJB9	1	−94.8	1793	4.407	10.079	−4.922
	2	−79.1	842	5.237	17.197	10.288
	3	−73.6	138	−8.823	10.663	5.216
	4	−66.8	47	−12.106	−3.433	−3.645
	5	−58.8	42	−7.031	16.08	11.506
MAPK9	1	−6.8	842	39.411	−20.462	−11.75
	2	−6.3	623	53.604	−25.892	−16.211
	3	−6.2	423	−6.78	−24.873	20.747
	4	−6.1	413	29.102	−27.891	0.132
	5	−5.9	350	39.576	−24.276	−56.974

## 4 Discussion

MPs refer to plastic fragments or particles smaller than 5 millimeters in diameter. They primarily enter the human body through three routes: inhalation (via the respiratory system), ingestion (via the digestive tract), and skin contact. As ubiquitous environmental pollutants, MPs significantly increase human exposure risks due to their small particle size and widespread distribution. In particular, inhalation of plastic fibers through the respiratory tract may pose potential threats to human health [39]. Research indicates that the toxic effects of MPs exhibit significant size dependence—smaller particle sizes possess larger specific surface areas and higher surface activity, making them more prone to adsorbing toxic organic pollutants and inorganic pollutants from the environment. These adsorbed substances can subsequently impact organisms through bioaccumulation or direct toxic effects [40]. In recent years, the association between microplastic exposure and respiratory toxicity has garnered increasing attention, potentially contributing to respiratory diseases by inducing oxidative stress, inflammatory responses, or epithelial cell damage [13]. However, existing research primarily focuses on general toxicological effects of MPs, with limited investigation into their specific respiratory injury mechanisms. Therefore, this study integrated multi-omics data with bioinformatics methods to identify three key targets (DNAJB9, SQSTM1, MAPK9). Their diagnostic efficacy in AR was validated through heatmaps and ROC curve analysis. Results indicate that these three targets are significantly downregulated in AR patients and highly correlated with disease risk. This suggests they may participate in microplastic-induced AR progression by regulating pathways such as apoptosis and autophagy.

Several factors contribute to the mechanism by which MPs invade the human body and cause tissue damage. Research by Mengyuan Liu’s team reveals that microplastic particles can migrate to respiratory epithelial tissues through diffusion, cell penetration, or endocytosis, subsequently being internalized by epithelial cells and entering the intracellular space [13]. Notably, the efficiency of microplastic internalization and its toxic effects are closely related to particle size, surface charge, and other physicochemical properties [41]. Halimu et al.’s findings indicate that smaller or positively charged microplastic particles exhibit faster internalization rates and more pronounced toxic effects compared to larger or negatively charged particles [42]. Beyond direct internalization, disruption of the epithelial barrier function represents another key pathway for microplastic entry into the body [13]. Existing studies reveal that in mouse models exposed to MPs, the expression level of tight junction protein ZO-1 in the airway epithelium is significantly downregulated, and trans-epithelial electrical resistance (TEER) is reduced, indicating compromised airway epithelial barrier integrity [43]. This barrier disruption is often accompanied by increased secretion of pro-inflammatory factors and activation of oxidative stress pathways. Mechanistic studies by Xu et al. demonstrated that MPs can be internalized into airway epithelial cells, stimulating secretion of heat shock protein 90α (HSP90α), which subsequently activates the PI3K/AKT/mTOR signaling pathway, ultimately leading to airway epithelial cell apoptosis and barrier dysfunction [44]. In summary, MPs, due to their minute particle size, can cause damage to human tissues at the cellular level through direct internalization and disruption of the epithelial barrier.

Additionally, immune system activation plays a crucial role in the microplastic-induced respiratory injury process. Enrichment analysis results from this study indicate that microplastic-induced AR injury is closely associated with immune regulatory mechanisms such as the IL-17 signaling pathway, Toll-like receptor (TLR) signaling pathway, and apoptosis. Previous studies have demonstrated that microplastic exposure significantly exacerbates the imbalance in Th1/Th2 immune responses [45,46]. Animal experiments demonstrate that microplastic exposure elevates cytokine levels (including IL-13, IL-10, IFN-γ, and TNF-α) in asthmatic mouse models and promotes total IgE secretion, thereby disrupting the Th1/Th2 cytokine balance [47]. Notably, Yang et al. found that while microplastic exposure elevated serum IgG1 levels in normal mice, it had no significant effect on IL-4 and IL-5 expression in asthma model mice [43]. Furthermore, no significant changes in IgE levels were observed in this study, potentially related to the specific microplastic type and the presence of additional additives. Furthermore, abnormal activation of Th17 cells can enhance Th2-mediated eosinophilic airway inflammation, potentially through dysregulation of the TTRPA1-p38 MAPK signaling pathway [46]. Collectively, microplastic exposure induces immune imbalance by shifting the response toward Th2-dominant immunity, while simultaneously exacerbating inflammatory progression in the presence of additives.

Toxicological analyses showed that the toxicity of MPs was associated with hERG blockers, eye irritation, and respiratory effects, and these effects were consistent with the pathogenic pathways of AR, confirming the important role of MPs in the progression of AR. The enrichment analysis of MPs affecting AR-related targets pointed to the “Regulation of Cell Death”, “Apoptotic Process” and other programmed death regulatory processes, and the results of the GSEA pathway showed that DNAJB9, SQSTM1, and MAPK9 were mainly enriched in “mTOR signaling pathway” and other processes. Differential expression analysis showed that these three targets were down-regulated in AR, and MPs may contribute to AR disease progression by negatively regulating programmed cell death.

The DNAJB9 gene encodes MRJ, a member of the heat shock protein 40 family, which is involved in protein folding, translocation, and degradation, and is essential for maintaining intracellular protein homeostasis [48]. Kim HY et al. found that ectopic repair of DNAJB9 inhibited migration, invasion, and lung colonization of triple-negative breast cancer cells, which stabilized the FBXO45 protein by inhibiting its ubiquitylation, and decreased the ZEB1 abundance, thereby inhibiting epithelial-mesenchymal transition (EMT) and metastasis [49]. In addition, DNAJB9 is involved in the degradation of cystic fibrosis-associated proteins as a tubulointerstitial co-chaperone and is a therapeutic target in cystic fibrosis nephritis [50]. However, the function of DNAJB9 in AR is unclear. In this study, DNAJB9 was found to be down-regulated in AR and enriched in the “mTOR signaling pathway”, etc. mTOR regulates cell growth, metabolism, and autophagy, and DNAJB9 may affect mTOR activity by modulating the folding and stability of related proteins. MPs may lead to the downregulation of DNAJB9, which promotes the proliferation of nasal mucosal epithelial cells and inhibits autophagy, thus contributing to the development of AR.

SQSTM1 (also known as p62) is an autophagy receptor protein that belongs to the activators of the non-classical KEAP1-NFE2L2/NRF2 pathway [51–53]. Because p62 accumulates when autophagy is inhibited and its level decreases when autophagy is induced, SQSTM1 can thus be used as a marker of autophagic flux [54]. SQSTM1 co-localizes with ubiquitylated protein aggregates in a variety of neurodegenerative disorders and hepatic proteinopathies [55–57]. However, the role of SQSTM1 in AR remains poorly understood. Liu N et al. identified differentially expressed proteins in the urine of AR patients by mass spectrometry proteomics and found that SQSTM1 was associated with protein structural domain-specific binding [58]. In the present study, SQSTM1 was down-regulated in AR and was enriched in important pathways such as the “phosphatidylinositol signaling system” and the “mTOR signaling pathway”. MPs may upregulate autophagy in the mTOR pathway through the downregulation of SQSTM1, thereby protecting nasal mucosal epithelial cells from the toxic stimulation of MPs and inhibiting the development of AR.

MAPK9 is a member of the MAPK family that encodes a kinase that plays a key role in cellular stress, gene transcription, cell growth, apoptosis, and neurodevelopment. [59]. Studies have shown that MAPK9 presents overexpression in a variety of tumors and promotes tumor progression. For example, a study by Wang Z et al. found that CircRNF20 stimulated the proliferation of non-small cell lung cancer by activating MAPK9 [60]. In addition, JinZe L et al. identified MAPK9 as a key gene related to iron death by bioinformatics methods and indicated that it may promote the progression of spinal cord injury [61]. Meanwhile, Yingying Z et al. found that MPs may induce micro mitochondrial autophagy and apoptosis in zebrafish intestinal epithelial cells through the Kras/MAPK signaling pathway [62]. However, the role of MAPK9 protein in AR has not been reported. However, the role of MAPK9 in AR has not been reported. The results of the present study indicate that MAPK9 is down-regulated in AR and is enriched in the processes of “basic transcription factor”, “β-alanine metabolism” and “spliceosome”. MPs may inhibit autophagy and promote apoptosis by down-regulating MAPK9, thus promoting the development of AR.

In a drug prediction and molecular docking study, we identified resveratrol as a natural small-molecule compound capable of interfering with the effects of MPs on AR. It has been shown that resveratrol can attenuate the toxic response of MPs in humans. For example, Xingpei F et al. found that treatment with the antioxidant resveratrol inhibited ROS-induced activation of the NFκB-MAPK signaling pathway in mice by constructing a nanoplastic mouse model, which in turn ameliorated nanoplastic-induced glucose and lipid metabolism disorders [36]. Shiwakoti S et al. also found that, in the polystyrene nanoplastic swine model resveratrol was able to attenuate polystyrene-induced endothelial senescence and dysfunction [38]. In addition, many studies have demonstrated the potential role of resveratrol in the treatment of allergic rhinitis, as it can effectively alleviate AR in mice by inhibiting the oxidative stress pathway of TXNIP, and its mediated activation of SIRT1 also attenuates ovalbumin-induced ARin mice [63]. Therefore, resveratrol can be used as a promising natural small-molecule compound for the treatment of the toxic effects of MPs on AR.

In summary, the findings of this study provide a theoretical basis for the health hazards posed by microplastics and offer a potential new therapeutic target for allergic rhinitis, facilitating the development of more effective treatments or interventions. However, this research primarily relies on network analysis and molecular docking simulations, lacking validation through in vivo and in vitro experiments. Further experimental studies are required to validate these findings. Additionally, given the wide variety of microplastics, future research should be expanded to include more types of microplastics.

## 5 Conclusion

This study investigated the toxic mechanisms by which MPs induce AR through ADMETlab toxicity assessment, multi-omics target screening, and functional enrichment analysis. Three core targets—DNAJB9, SQSTM1, and MAPK9—were identified as key regulators of AR progression by modulating critical biological pathways including apoptosis and autophagy. Simultaneously, it confirmed that resveratrol exhibits strong binding affinity with these three targets, demonstrating molecular potential as a therapeutic agent for microplastic-related AR. This study provides molecular mechanistic evidence for environmental aetiology research on microplastic-related allergic rhinitis.

## Supporting information

S1 Table All the original data in the study. (XLSX)

S2 Table Intersection of microplastic toxicity targets and differentially expressed genes in allergic rhinitis. (DOC)
