# Correction: Microplastic exposure and allergic rhinitis: Network toxicology, and molecular docking insights

**DOI:** 10.1371/journal.pone.0339701

**Published:** 2025-12-29

**Authors:** Yaojun Wang, Dandan Xu

The affiliation #1 was incorrectly assigned to the second author. Dandan Xu should be listed as affiliated only with #2 and #3. The correct affiliations are: 2 Central Laboratory, The First Affiliated Hospital of Hebei North University, Zhangjiakou, China. 3 Hebei Key Laboratory of Systems Biology and Gene Regulation, The First Affiliated Hospital of Hebei North University, Zhangjiakou, China.
