# Supplementary material for: Microplastic exposure and allergic rhinitis: Network toxicology, and molecular docking insights
Source: PLoS One. 2025 Oct 17;20(10):e0334162. doi: 10.1371/journal.pone.0334162 (PMC12533909; doi:10.1371/journal.pone.0334162)
Supplement: S2 Table — (DOC) [file pone.0334162.s002.doc]

Supplementary Table S2 Intersection of microplastic toxicity targets and differentially expressed genes in allergic rhinitis

| Crossgenes | logFC |
| --- | --- |
| DHCR24 | 0.711 |
| ANGPT1 | -0.0168 |
| ACVRL1 | 0.0047 |
| MAP1LC3B | -0.431 |
| OPA1 | -0.524 |
| DNAJB9 | 0.726 |
| SQSTM1 | -0.649 |
| CASP8 | -0.00667 |
| SDAD1 | 0.288 |
| ID1 | -0.784 |
| DBF4 | -0.00863 |
| SDF2L1 | 0.691 |
| TOR3A | 0.987 |
| MAPK9 | -0.468 |
| CCND3 | -0.279 |
